# The contribution of online content to the promotion and normalisation of female genital cosmetic surgery: a systematic review of the literature

**DOI:** 10.1186/s12905-015-0271-5

**Published:** 2015-11-25

**Authors:** Hayley Mowat, Karalyn McDonald, Amy Shields Dobson, Jane Fisher, Maggie Kirkman

**Affiliations:** Jean Hailes Research Unit, Monash University, Melbourne, Australia; Institute for Advanced Studies in the Humanities, University of Queensland, Brisbane, Australia

**Keywords:** Female genital cosmetic surgery, Systematic review, Internet, Labiaplasty, Women’s health

## Abstract

**Background:**

Women considering female genital cosmetic surgery (FGCS) are likely to use the internet as a key source of information during the decision-making process. The aim of this systematic review was to determine what is known about the role of the internet in the promotion and normalisation of female genital cosmetic surgery and to identify areas for future research.

**Methods:**

Eight social science, medical, and communication databases and Google Scholar were searched for peer-reviewed papers published in English. Results from all papers were analysed to identify recurring and unique themes.

**Results:**

Five papers met inclusion criteria. Three of the papers reported investigations of website content of FGCS providers, a fourth compared motivations for labiaplasty publicised on provider websites with those disclosed by women in online communities, and the fifth analysed visual depictions of female genitalia in online pornography. Analysis yielded five significant and interrelated patterns of representation, each functioning to promote and normalise the practice of FGCS: *pathologisation of genital diversity; female genital appearance as important to wellbeing; characteristics of women’s genitals are important for sex life; female body as degenerative and improvable through surgery;* and *FGCS as safe, easy, and effective*. A significant gap was identified in the literature: the ways in which user-generated content might function to perpetuate, challenge, or subvert the normative discourses prevalent in online pornography and surgical websites.

**Conclusions:**

Further research is needed to contribute to knowledge of the role played by the internet in the promotion and normalisation of female genital cosmetic surgery.

## Background

There is increasing evidence that women are pursuing surgical modification of the vulva for cosmetic reasons. Popular surgical procedures include, but are not limited to, reduction of the labia minora and clitoral hood, tightening of the vagina, ‘plumping’ of the labia majora, liposuction of the mons pubis, and ‘G-spot’ collagen injections [1, 2]. In Australia, data captured by Medicare, the national publicly-funded universal healthcare insurance scheme, indicate that the number of claims for labia reduction increased threefold in the decade from 2001 – 2010 [3]. These data do not capture the total number of labiaplasties occurring across the country because medical necessity must be demonstrated in order to claim surgical costs under the Medicare scheme; procedures sought without medical indication are paid for privately [4, 5] and are not recorded in national registers.

The apparent popularity of female genital cosmetic surgery (FGCS) procedures in recent years has triggered a flurry of critical academic engagement in the topic. Previous empirical research has shed light on a range of aspects relevant to the phenomenon, from factors that may influence the decision of individual women to seek FGCS [5] to mainstream media representations of female genitalia [6, 7]. Other scholars have sought to theorise the complex ethical debates around the practice of FGCS, specifically in relation to the heavily critiqued practice of female genital cutting/mutilation [8–10]. The focus of such debates, and subsequently this review paper, is not the use of surgery to address functional difficulties but the rise in demand for aesthetically-driven procedures and the extent to which the distinction between the two has become increasingly blurred. In the absence of formal, standardised medical indications for these procedures [11], the identification of ‘pathological’ and ‘normal’ is subjective. Plastic surgeons are significantly more likely than other physicians to regard larger labia minora as “distasteful and unnatural”, male surgeons more so than their female counterparts [12].

There is evidence that women’s external genitalia are highly diverse [13]. However, the practice of FGCS appears to be underpinned by a desire for a particular homogenous genital aesthetic, namely a “tight” vagina [14] and “clean slit” [15, 16] or “Barbie Doll” [7, 17] vulva, in which the labia minora are not visible. The normalisation of these ideals has been linked to various potential influences, including the popularity of pubic hair removal [17], long-held negative societal attitudes toward female genitalia [6, 18], and limited aesthetic diversity in magazines, both mainstream and pornographic [7, 19]. Bramwell’s [6] analysis of women’s magazines found that the vast majority of images depicted the female pubic area as flat or a smooth curve. Of the 8 % of images that showed any indentations or extrusions, such detail was attributable to the bunching of clothing rather than genital detail [6]. Schick and colleagues [7] noted a similar trend amongst Playboy Magazine centrefolds, reporting that none of the images portrayed prominent labia minora, nor did they represent realistic colour variation, with genitalia uniformly portrayed as pink or pale red.

Whereas traditional media have long been understood to influence female beauty ideals [20] and attitudes towards cosmetic surgery [21], recent studies suggest that the internet plays a larger role. For example, Walden and colleagues [22] found the internet to be an important tool in the decision-making process for women considering breast augmentation. Given that many women are reluctant to discuss concerns about their genitalia with health care practitioners [23, 24], the anonymity afforded by the internet may make it an even more powerful reference point for those considering genital surgical modification. Indeed, a recent study found the internet and pornography to be the two major media influences on consideration of labiaplasty among Australian women [25]. Internationally, Michala and colleagues [26] found that the internet was a primary source of information for older adolescents presenting for labiaplasty in their Greek sample. Further, a survey conducted in The Netherlands found that women who used the internet as their source of information about labia reduction surgery assessed the procedure as more acceptable and considered having the surgery more often than women who obtained information from other sources, such as mainstream media, peers or a physician [27]. In light of these findings, and the pervasiveness of online marketing of FGCS, it is important to gain a better understanding of the information that is circulating in the online sphere about female genitalia and female genital cosmetic surgery. This systematic review was designed to do so by synthesising existing research on female genital cosmetic surgery and the internet.

## Method

### Inclusion criteria

Papers were eligible for inclusion if they were published before September 2014, in English, in peer-reviewed journals, and reported empirical research (using qualitative or quantitative methods) about online representations of female genital appearance or female genital cosmetic surgery.

### Search strategy and selection of papers

Eight social science, medical, and communication databases (Medline, CINAHL, ProQuest, Scopus, Sociological Abstracts, Social Science Citation Index, Communication and Mass Media Complete, and PsycInfo) were individually searched using the terms “*social media*” OR “social network*” OR *internet* OR *youtube* OR *twitter* OR *tumblr* OR *facebook* OR “*online communities*” OR “*bulletin board*” OR web* OR *wiki* OR blog* OR *email;* in conjunction with: genital* OR labia* OR vulv* OR vagin* OR clitor* OR hymenop*. Where applicable, the relevant subject terms for each database were also included. Google Scholar was searched using the terms “female genital*”, *labiaplasty*, *internet*. Reference lists of located articles were hand-searched for other potentially relevant titles.

Following the removal of duplicates, records were assessed for suitability in accordance with the PRISMA guidelines [28], first by title, then by abstract. To determine final inclusion, articles were obtained and read in full.

### Assessment of quality

Given that all eligible papers were likely to report analysis of texts or images, we sought standardised criteria, tools, or frameworks to use in assessing their quality, but found nothing designed for the purpose. Two authors (HM and KM) independently assessed the quality of each study using Kmet’s [29] assessment tool for quantitative and qualitative studies. Discrepancies were discussed among three authors (HM, KM, and MK) and resolved. The quantitative and qualitative checklists comprise, respectively, 14 and 10 questions (see Tables 2 and 3), each of which assesses against a criterion. If the item meets the criterion it is scored 2, if it partially meets the criterion it is scored 1, and if it fails to meet the criterion it is scored 0. If a criterion is assessed as “not applicable”, 2 points are deducted from the final total (maximum possible = 28). Calculated scores were defined as strong (>80 %), good (70–80 %), adequate (50–70 %), or limited (<50 %).

### Data analysis

Results reported in all papers were analysed thematically. As themes were identified in each paper, a structure of themes and sub-themes was developed. Each paper was reassessed against the developing thematic structure to ensure the best fit and to establish relationships among themes and concepts. In addition to the results reported and conclusions drawn by the authors of each paper, reviewers used all identified themes in analysing the content of any included data excerpts, in order to achieve the most comprehensive account of the literature.

## Results

**Fig. 1:**
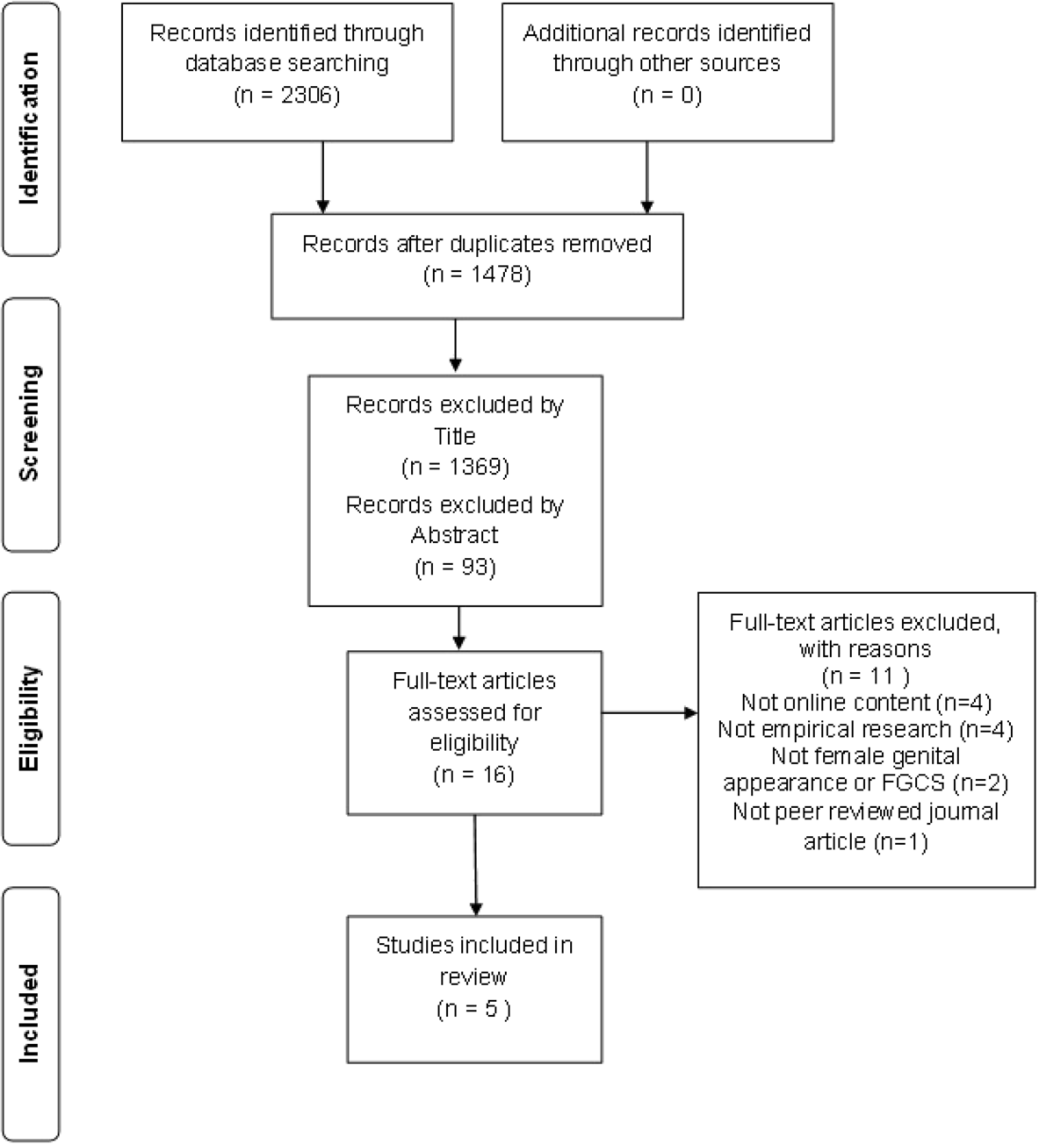
Flow diagram of study selection process, based on the PRISMA statement [28]

The search strategy yielded five eligible papers, each reporting on a single study. Details of the selection process are outlined in Fig. 1. A summary of papers is presented as Table 1.

**Table 1:** Summary of reviewed papers

Author Year Country of Origin	Aim	Method	Sample	Quality rating, limitations
Ashong & Batta 2012 [34]	To explore the content of Western female genital cosmetic surgery provider websites.	Qualitative. Textual analysis, methods not described in detail.	11 international FGCS provider websites (6 USA , 2 UK, 1 Canada, 1 Belgium, 1 Brazil). Does not specify which pages from each site were analysed.	Limited (8/20): lack of rigorous data analysis or theoretical connection.
Nigeria				
Howarth, Sommer & Jordan 2010 [31]	To determine if visual depictions of female genitalia differ across 3 sources (online pornography, anatomy textbooks, and feminist publications: online, print).	Quantitative. Comparison of measurements of vulval features from screen and book photos or illustrations.	253 images (98 from 3 free online pornography websites, 29 from <92 human anatomy textbooks, 126 from feminist publications: 2 books, 1 website).	Strong (18/22): limited by unclear sampling strategy and failure to differentiate sources of individual images.
United Kingdom, The Netherlands				
Liao,Taghinejadi & Creighton 2012 [32]	To investigate the clinical information on female genital cosmetic surgery provider websites.	Quantitative. Content analysis.	10 international FGCS provider websites (5 UK, 5 USA).	Strong (18/18).
United Kingdom				
Moran & Lee 2013 [33]	To examine how the textual and visual content of the Australian labiaplasty provider websites normalises the practice of FGCS.	Qualitative. Multimodal critical discourse analysis.	4 Australian FGCS provider websites: all textual and visual content on home pages, (cosmetic surgery in general), and content related to labiaplasty from entire site.	Strong (18/20): limited by only partial use of verification procedures and reflexivity.
Australia				
Zwier 2014 [30]	To compare motivations for considering labiaplasty expressed by women on online communities with those indicated on the websites of an international sample of surgery providers.	Quantitative. Content analysis.	40 international English- or Dutch-language FGCS provider websites (Australia, Canada, Ireland, the Netherlands, New Zealand, South Africa, UK, USA). 78 posts in which women (28 Dutch, 25 US, 25 UK) wrote about their reasons for labiaplasty, drawn from 4 online communities with recent threads about labiaplasty (1 Netherlands, 2 USA, 1 UK). Ages disclosed by posters ranged from 12 to 61 years.	Strong (21/22): limited by inconsistency of sample. A selection of Dutch, US and UK websites would have enabled more accurate comparison between content of online communities and websites.
The Netherlands				

Four of the five studies were conducted by researchers based in high-income Western countries: the Netherlands [30, 31], the United Kingdom [31, 32], and Australia [33]. The remaining study was conducted in a low-income country, Nigeria [34]. Four studies explored internet content that had been produced across multiple countries in the Anglophone West [30–33], reflecting both the globalised nature of the internet and also that this particular form of genital surgery may be prominent in Westernised societies and cultures. One paper reported research of sites based in a single country (Australia) [33].

Liao and colleagues [32] report results of a content analysis of 10 FGCS provider websites, based in the United States and United Kingdom, with a specific emphasis on the breadth, depth, and quality of clinical information provided. Moran and Lee [33] limited their sample to four websites belonging to Australian labiaplasty clinics but used the techniques of multimodal critical discourse analysis to examine how both the textual and visual content of the sites contributed to the normalisation of FGCS. Writing from Nigeria, Ashong and Batta [34] analysed the textual content relating to FGCS on 11 websites for FGCS provider clinics based in the United States, the United Kingdom, Belgium, Canada, and Brazil. The authors were particularly interested in integrity of the information supplied, and the overall trend towards the commercialisation of female genitalia through medicine, arguing that “the hype surrounding cosmetic or aesthetic genital surgery is a damaging distraction particularly when [Africa] is waging a battle against female genital mutilation” [34]. Zwier’s [30] paper reported the only study of social media content, comparing motivations for labiaplasty disclosed by 78 women posting in online communities with those publicised by 40 labiaplasty provider websites. In a report of the sole study to examine online representations of female genitalia without specific reference to FGCS, Howarth and colleagues [31] compared visual depictions of female genitalia in online pornography to those depicted in anatomy textbooks and feminist publications (print and online) to assess how patterns of representation might influence public perceptions of ‘normality’.

### Quality assessment

Tables 2 and 3 provide summaries of the quality assessment of reviewed papers and the rating assigned to each paper. Four of the five articles were rated as of Strong quality. Despite assessing the quality of Ashong and Batta’s [34] paper as Limited, we chose to include it for two reasons. First, there is a scarcity of African perspectives in Western academic debates about FGCS, a gap that is particularly pertinent given the parallels between FGCS and female genital cutting/mutilation. Further, their findings were consistent with those reported in the other four papers.

**Table 2:** Quality Assessment Matrix, Quantitative Studies [29]

Study	Howarth, Sommer & Jordan [31]	Liao, Taghinejadi & Creighton [32]	Zwier [30]
Question/objective sufficiently described?	Yes	Yes	Yes
Study design evident and appropriate?	Yes	Yes	Yes
Method of subject/comparison group selection described and appropriate?	Partial	Yes	Partial
Subject (and comparison group) characteristics sufficiently described?	Partial	Yes	Yes
Outcomes well defined and robust to measurement/misclassification bias? Means of assessment reported?	Yes	Yes	Yes
Sample size appropriate?	Partial	Yes	Yes
Analytic methods described/justified and appropriate?	Yes	Yes	Yes
Some estimate of variance is reported for the main results?	Yes	N/A	Yes
Controlled for confounding?	Partial	N/A	Yes
Results reported in sufficient detail?	Yes	Yes	Yes
Conclusions supported by the results?	Yes	Yes	Yes

**Table 3:** Quality Assessment Matrix, Qualitative Studies [29]

Study	Ashong & Batta [34]	Moran & Lee [33]
Question/objective sufficiently described?	Yes	Yes
Study design evident and appropriate?	Partial	Yes
Context for the study clear	Yes	Yes
Connection to a theoretical framework/wider body of knowledge	Partial	Yes
Sampling strategy described relevant and justified	Partial	Yes
Data collection methods clearly described and systematic	Partial	Yes
Data analysis clearly described and systematic	No	Yes
Use of verification procedures to establish credibility	No	Partial
Conclusions supported by the results?	No	Yes
Reflexivity of the account	No	Partial

### Themes

Five interrelated themes were identified. These were: *pathologisation of genital diversity* (identified in 5 papers), *female genital appearance as important for wellbeing* (4 papers), *characteristics of women’s genitals as important for sex life* (4 papers), *the female body as degenerative and improvable through surgery* (3 papers), and *FGCS as safe, easy, and effective* (3 papers).

#### Pathologisation of genital diversity

All studies found that vulval diversity is pathologised in cyberspace, with the concomitant promotion of a homogenised “clean slit” [15, 16] vulva as ideal and desirable. Pathologisation occurred textually, with providers using medical terminology and disparaging language to position certain characteristics as abnormal or undesirable, and through visual representations on these websites and in online pornography.

Three papers reported FGCS surgical provider websites employing a range of disparaging language to signify which genital characteristics or features ought to be considered undesirable. For instance, pubic fat is described as “unsightly”, vaginas as potentially “loose”, and large labia as “protuberant”, “oversized”, “awkward”, and “unappealing” [32–34]. In addition to such overtly pejorative descriptions, Liao and colleagues [32] noted that sampled FGCS provider websites made latent associations between larger labia, ugliness, and poor personal hygiene.

Findings from four studies support the conclusion that vulval diversity is pathologised by the presentation of certain characteristics as unnatural or diseased. Two studies identified FGCS provider websites using medical terminology to construct protruding labia minora, amongst other genital characteristics, as pathological and thus requiring surgical correction [32, 33]. A third paper quoted excerpts from surgical websites in which such medical rhetoric was visible, but this was not specifically addressed or interrogated by the authors [34]. Liao and colleagues [32] and Moran and Lee [33] noted that, although terms such as ‘labial hypertrophy’ may appear to the lay consumer to refer to established medical conditions, they lack formal scientific definition. In particular, in the case of ‘labial hypertrophy’, Liao and colleagues [32] suggest that the lack of specificity allows surgeons to apply the label to women without any clinical indication. Three papers found provider websites proffering potential causes of these supposedly undesirable features, such as ageing, childbirth, or weight gain [32–34]. It was noted that a discussion of causes further positions genital variations outside the limited ideal as abnormalities or defects in need of surgical correction [33].

The pathologisation of vulval diversity does not occur solely through language. The findings reported in all five papers indicate that online visual representations of female genitalia can be a powerful indicator of socially desirable and undesirable genital characteristics. Four studies reported the presence of before-and-after labiaplasty image galleries on FGCS provider websites, with the frequency ranging from 25 to 60 % of the websites in each sample [30, 32–34]. Two papers reported detailed analysis of these images, with authors concluding that the images serve to pathologise natural vulval diversity by contrasting ‘before’ examples, all of which fell within the range of labial dimensions observed in the female population, with standardised ‘after’ images of vulvas with no visible labia minora [32, 33].

Howarth and colleagues [31] found the range of female genitalia depicted in three popular free online pornography websites (*N* = 98 images) to be significantly less protuberant and less varied than the range found in the female population. The authors also analysed a sample of images drawn from feminist-oriented publications (one website and two books) (*N* = 126 images) which they considered to be a closer reflection of the range of variation amongst women [31]. However, because the authors did not make any distinction in this sub-sample between the images sourced online and those from print publications, we cannot draw any definitive conclusions about the range of female genitalia depicted on this website.

#### Female genital appearance as important for wellbeing

Results from four studies indicate a strong discursive connection between female genital appearance and wellbeing. This connection was often drawn by commercial FGCS providers in order to promote their services but was also supported and reinforced by women’s own accounts of their motivations and experiences. Four papers identified FGCS provider websites drawing an unquestioned connection between female genital appearance and psychological or emotional wellbeing or distress [30, 32–34]. Specifically, provider websites were found to suggest that female genitals that are deemed to be aesthetically unpleasing cause a woman shame and embarrassment, leading to “devastating effects on her life” [34].

Zwier’s [30] analysis found that 98 % of provider websites promoted FGCS as a solution to emotional discomfort such as feeling “freakish” or ashamed about one’s genital appearance. Indeed, it was reported that FGCS websites were more likely to emphasise surgery as a solution to emotional problems than to physical pain or functional issues [30]. Liao et al. [32] found 100 % of provider websites in their sample suggested several “social and psychological advantages” of a modified vulval appearance through FGCS, such as improved self-confidence and a “sense of freedom”.

Using the techniques of critical discourse analysis, Moran and Lee [33] interrogated this connection, concluding that surgical websites frame emotional or psychological distress as an inevitable by-product of possessing certain genital characteristics, rather than a societal failure to recognise such variation as natural or acceptable. For example, websites claimed that “suitable candidates for labial rejuvenation surgery” are women “who experience psychological distress due to appearance of their labia” and that “enlarged or exposed labia causes much stress about the appearance of the inner and outer lips of the vagina” [33]. The authors also draw attention to the subtle ways in which this connection is reinforced through common idioms on surgical websites, as women reviewing the sites are encouraged to care for their “skin, body and soul”, “optimise your health, wellbeing and appearance”, and to “look and feel your very best” [33].

Zwier’s [30] analysis of women’s accounts of motivations for FGCS, as disclosed in online communities, suggests that this connection may have significant cultural resonance with women. This was the only study among the reviewed papers to explore user-generated content. The author found that emotional discomfort is the most commonly cited reason for desiring or pursuing FGCS, discussed by 71 % of women; 42.5 % of women gave this as their sole reason, with the remainder alluding to additional motivations such as discomfort in tight clothing or when exercising [30]. Zwier [30] reports the intense emotions communicated by women in their online contributions; for instance: “I hate mine, hate, hate HATE it”.

Notably, two of the websites quoted in Ashong and Batta’s [34] study suggested that the distress was related to how a woman “perceives” her genital appearance, a construction of the problem that was not evident in the other papers reviewed. However, regardless of whether the emotional distress is attributed to objective assessment of genitalia or is mediated by the woman’s perception, all four analyses of FGCS provider websites found the content of these sites to position FGCS as the logical solution to emotional distress, with the sites asserting that surgery will “restore” and “enhance” the self-confidence of women who undergo it [30, 32–34].

#### Characteristics of women’s genitals are important for sex life

Findings from four papers indicate that FGCS provider websites commonly claim associations between pre-operative genitalia and sexual dysfunction, with the dysfunction described as primarily psychological [30, 32–34]. Although sexual dysfunction is attributed, in some cases, to pain and discomfort, provider websites and women’s online accounts both designate the primary source of sexual dysfunction as shame, embarrassment, and fear of adverse reactions from male sexual partners [30, 32–34]. FGCS provider websites assert sexual dysfunction and thus that women’s sex lives will be improved by undergoing labiaplasty or other genital cosmetic procedures [32, 33].

Although it might be inferred that a woman’s sexual satisfaction is being prioritised, three studies found that FGCS provider websites promoted surgery as the solution to a male partner’s sexual dissatisfaction [32–34]. In some instances, this was achieved by highlighting the reasons for potential dissatisfaction, with websites making claims such as “the loose and unsatisfying feeling that women feel can also be felt by their male partner during intercourse” [34]. In others, it is stated that “the sexual partner will feel a difference after labiaplasty”, or “will clearly notice this change for the better” [33]. Liao and colleagues [32] reported that some provider websites recommended FGCS to benefit intimate relationships in general because it improved interpersonal “disharmony and resentment”. These assertions are reflected in the anxieties evident in the women’s posts in online FGCS communities, with 37.5 % reporting fear of negative reactions from sexual partners as a motivation for labiaplasty [30]. A further 11 % wrote that they expected their sexual enjoyment to be enhanced by the procedure [30].

#### The female body as degenerative, improvable through surgery

Having established that a “tight” vagina and a “tidy”, “youthful” vulva is represented online as the (Western) ideal, three papers reported online content that contributes to a cultural representation of a tenuous female body susceptible to degeneration, particularly through childbirth and ageing, and improvable or restorable through surgery [32–34]. Ageing or post-baby bodies are pathologised through descriptors such as “loose”, “descending” or “hanging”, all terms that carry unflattering connotations in a society that valorises youth [33, 34]. Provider websites recruit sexual partners to reinforce the need to reverse genital deterioration by suggesting that they will notice and dislike intimate parts of her body, visible only to them: “a woman might have a face lift and look really young until she goes to bed and a partner can see the evidence of ageing there” [32]. Three papers note that FGCS provider websites tend to depict almost exclusively young, slim, Caucasian women [32–34].

Several surgery clinics are reported as promoting “Mommy Makeover” packages, in which vaginal tightening and labia reduction surgeries are bundled together with liposuction, tummy tucks, and breast augmentations [32, 33]. These packages promise to restore a woman’s “previously gorgeous body” [33]. The authors argue that these promotional activities reframe the physical effects of bodily processes such as pregnancy and childbirth as undesirable conditions necessitating reversal through medical intervention [33].

#### FGCS as safe, easy and effective

It was reported in papers from three studies that FGCS provider websites claimed benefits and asserted the safety of various FGCS procedures, without providing evidence [32–34]. Liao and colleagues [32] found that provider websites tended to emphasise non-specific social and psychological advantages, such as improved self-confidence, relief of discomfort, and better hygiene. Where specific medical claims were made, these were not supported by reference to any clinical evidence. Such claims included the assertion that G-spot injections “revolutionise many women’s sex lives” and that, in the case of labiaplasty, “sensation may even be enhanced because of the new nerve endings and removal of the tissue” [32].

Two papers reported on the success rates published on surgery provider websites, finding that, where cited, these were in the range of 90 – 95 % [32, 33]. Liao and colleagues [32] found that other sites boasted “an excellent track record” or “the best results worldwide”. Two website studies reported on the use of personal testimonials on surgeon websites, with Liao and colleagues [32] finding flattering personal testimonials on 30 % of the websites in their study. Without similarly quantifying what they found, Ashong and Batta [34] characterised the testimonials on their sample sites as overwhelmingly adulatory, praising the benefits of FGCS and the surgeon and downplaying any potential adverse outcomes.

It was found in three studies that providers used medical terminology to confer credibility on FGCS in general and to legitimate specific surgical techniques and procedures [32–34]. Half of all providers investigated by Liao and colleagues [32] aligned themselves with the invented field of “cosmetic gynaecology”. This was the only paper to include a detailed interrogation of such language, reporting the use of 72 different terms or labels for female genital cosmetic surgery procedures across 10 provider websites [32].

Authors of three papers expressed concern about risk information on FGCS provider websites, arguing that potential risks or adverse outcomes of surgery are minimised or omitted entirely from website content [32–34]. Liao and colleagues [32] found that, although all sampled sites mentioned risk in some form, risks were either not specified (40 %) or limited to a list of standard surgical risks (60 %). Recovery expectations were idealised, with websites advising potential patients to expect “mild discomfort and swelling” and scars that will “disappear completely after 1 – 2 weeks” [34]. One paper reported that only two of the 10 sites studied mentioned scarring as a potential risk [32].

Information about potential surgical complications was almost exclusively in the context of warnings against “botched jobs” elsewhere, such as, “we have seen many unfortunate examples of terrible scarred uneven results of labiaplasty from other physicians” [32]. Accompanying claims were made that there are “no complications or side effects with any of our patients”[32]. Of 25 sites analysed by three papers, only one website was reported as citing a revision rate for its own clinic, giving a rate of 2 % for vaginal surgery [32].

## Discussion

This systematic review examined findings from five studies exploring online content relating to female genital appearance or female genital cosmetic surgery. Four of the five studies analysed the content of FGCS provider websites, and the consistency of their findings reveal the ways in which providers of female genital cosmetic surgery are promoting and normalising the practice of FGCS online. The most prominent theme, the pathologisation of vulval diversity, was also found in an analysis of online pornography. The results of this review suggest adverse implications for the women accessing these sites and reveal sociocultural attitudes to female genitalia and FGCS.

On female genital cosmetic surgery websites, the female body is pathologised by the medicalization and denigration of the aesthetic appearance of certain vulval features. Despite research indicating no significant association between pregnancy, childbirth, or natural ageing and vulval measurements [13], FGCS provider websites consistently assert that each causes undesirable deterioration that requires surgical intervention. The representation of the female body as degenerative, and improvable through surgery, sits within a broader Western culture in which youth is valorised, ageing pathologised, and concomitant self-surveillance and self-improvement are strongly encouraged [35, 36].

These websites perpetuate a persistent and unquestioned assumption that genital variation, beyond a restricted ideal, results in psychological and sexual dysfunction. We do not seek to discount the emotional distress and decreased quality of life experienced by some women because of internalised ‘self-loathing’ associated with the appearance of their genitalia. Indeed, the majority of women posting in online communities cite this as a motivation for surgery [30]. However, by discursively framing the distress as attributable to the genitalia themselves, rather than to a perceived failure to meet societal expectations [33], FGCS provider websites reinforce the ideal of the culturally constructed “clean slit” or “Barbie doll” vulva against which women must measure themselves [7, 15–17]. The deployment of such associations on provider websites fosters the very psychological and sexual distress for which FGCS procedures are recommended as solutions.

Further, the construction of certain natural genital variations as objectively abnormal and undesirable enables ‘corrective’ surgery to be positioned as the logical and empowering solution for affected women. Not only is surgery posited as the best solution to distress about one’s genitals, it is also promoted as an expression of personal agency and empowerment, a representation that is highly consistent with consumerist, neo-liberal, and post-feminist discourse more generally [33, 37]. This discourse functions in conjunction with the other themes identified in the review papers, particularly the pathologisation of diversity and the connection of FGCS to emotional wellbeing. After all, if certain genital features are objectively pathological and undesirable, and if correcting these through surgery is safe, easy, and beneficial to psychological health, then the decision to undergo surgery is neither superficial nor self-indulgent. Despite addressing what are primarily aesthetic concerns, FGCS is repositioned as a matter of reclaiming one’s self-confidence, life, and happiness, effectively distancing it from the critiques of vanity so commonly levelled at the practice of cosmetic surgery more generally [38]. The themes identified by this review are largely consistent with Braun’s [39] discussion, published as a book chapter and therefore ineligible for review. Braun analysed 20 FGCS provider websites and found various ways in which particular genital features and practices were pathologised or valorised; she concluded that the content of such sites was “deeply problematic” for women [39].

In line with Braun’s [39] critiques, the studies in this review found that the descriptions of sexual pleasure and dysfunction on FGCS provider websites were heteronormative and androcentric, focusing almost exclusively on heterosexual vaginal intercourse and the expectation that a tighter vagina and particular vulval aesthetic will result in increased sexual pleasure for both parties. The depiction of diverse, healthy female genitalia as problematic for male sexual partners and of FGCS as the only solution (as opposed to amending sexual activities, changing partners, or male-oriented surgery) reinforces an age-old view of passive female sexuality, in which women exist solely to appease the sexual desires of men [40]. That women are encouraged to surgically remove densely-innervated genital tissue only affirms this model.

A primary critique of FGCS is the extent to which these procedures are being performed in the absence of any clinical data on safety or long-term effectiveness [11]. This review found that many confident claims are being made across FGCS provider websites. In lieu of supporting evidence, providers foreground testimonials and, in some cases, assert high success rates without any indication of how these have been measured. Further, complications or patient dissatisfaction are positioned as the result of individual incompetence, not because surgeons may be poorly trained, the procedures lack evidence of safety and effectiveness, and the field as a whole is unregulated [41, 42].

## Conclusion

This body of literature, albeit small, draws attention to significant and interrelated patterns of online representation that function to pathologise natural genital variation and promote and normalise a homogenous, surgically altered ideal vulva to Western women. Although these studies have been able to discern and highlight particular patterns of representation, it should be acknowledged that such representations are open to interpretation, which will largely be influenced by individual viewer characteristics. Studies suggest that online content, including pornography and surgical websites, influences women’s consideration and acceptance of FGCS [25, 27]. Further qualitative inquiry into how women are using and interpreting such websites is warranted.

The majority of studies in this review analysed solely ‘non-social’ media: surgical websites and online pornography. However, the internet is not all direct marketing and pornography. Rather, the key feature or benefit of the internet is its capacity for diverse many-to-many communication opportunities. We speculate that this capacity may give people using social media the power to challenge dominant representations and discourses in cyberspace. Nevertheless, the sole study of espoused motivations for labiaplasty in online communities demonstrates that many women appear to have internalised derogatory patriarchal connotations of the vulva to an extent that adversely affects their lives and wellbeing [30]. In order to gain a fuller understanding of the role of the internet in the promotion and normalisation of FGCS, a detailed exploration of where and how individuals contribute to online discussions around female genital appearance and female genital cosmetic surgery would be valuable.

## Abbreviations

FGCS: Female genital cosmetic surgery
